# Review by urological pathologists improves the accuracy of Gleason grading by general pathologists

**DOI:** 10.1186/s12894-015-0066-x

**Published:** 2015-07-23

**Authors:** Yasushi Nakai, Nobumichi Tanaka, Keiji Shimada, Noboru Konishi, Makito Miyake, Satoshi Anai, Kiyohide Fujimoto

**Affiliations:** Department of Urology, Nara Medical University, 840 Shijo-cho, Kashihara, Nara 634-8522 Japan; Department of Pathology, Nara Medical University, 840 Shijo-cho, Kashihara, Nara 634-8522 Japan

**Keywords:** Gleason score, Prostate biopsy, General pathologist, Urological pathologist

## Abstract

**Backgrounds:**

Urologists use biopsy Gleason scores for patient counseling, prognosis prediction, and decision making. The accuracy of Gleason grading is very important. However, the variability of Gleason grading between general pathologists cannot be overlooked. Here we evaluate the discrepancy in the Gleason grading between 2 urologic pathologists and general pathologists as well as improvement in the accuracy of Gleason grading by general pathologists as a result of review by urologic pathologists.

**Methods:**

The subjects enrolled in the study were 755 patients who underwent prostate needle biopsy at affiliate hospitals of Nara Medical University over a period of 2 years. The biopsy samples were diagnosed by general pathologists. All biopsy samples were sent to Nara Medical University where they were diagnosed by 2 urologic pathologists. The results were then returned to the general pathologists. We compared the diagnostic accuracy of the general pathologists with that of the urologic pathologists for the parameters of no malignancy, atypical small acinar proliferation, high grade prostatic intraepithelial neoplasia and Gleason score (6, 3 + 4, 4 + 3 and 8–10). We then evaluated the concordance rate between the general and urologic pathologists for each of four consecutive 6-month periods.

**Results:**

The overall concordance rate of urologic pathologists and general pathologists in the first, second, third and last 6-month periods was 71.8 % (140/198), 79.8 % (168/225), 89.7 % (166/185) and 89.9 % (133/148), respectively. The concordance rate of the Gleason score between urologic pathologists and general pathologists in the first, second, third and last 6-month periods was 47.5 %(38/80), 62.6 %(57/91),76.9 %(50/65) and 78.7 %(48/61), respectively, and the kappa value was 0.55, 0.68, 0.81 and 0.84, respectively. The concordance rate improved significantly over the course of each period (*P* = 0.04).

**Conclusion:**

The concordance rate of the Gleason grading between the general pathologists and the urologic pathologists was 47.5 %. However, improvement of the concordance rate as a result of review by the urological pathologist could be seen.

## Backgrounds

Gleason et al [1] introduced the Gleason grading system for prostate cancer in 1966 and it was modified in 1974 [2] and 1977 [3]. Gleason grading is now accepted as the international standard for pathological grading in prostate cancer. In Japan, Gleason grading was introduced for clinical and pathological studies of prostate cancer in 2001; since then, it has been the standard for the pathological classification of prostate cancer [4]. Previously reported studies demonstrated the ability of the Gleason grading system to serve as a predictor of the final pathological stage and prognosis [5–7]. Generally, urologists use biopsy Gleason scores (GS) for patient counseling, prognosis prediction, and decision making. It goes without saying that the accuracy of Gleason grading is very important; however, several studies have described interobserver variabilities [8, 9]. The variability of Gleason grading between general pathologists cannot be overlooked [8, 10]. To improve these variabilities, the International Society of Urological Pathology (ISUP) convenced a consensus conference on the Gleason grading of Prostatic carcinoma at 2005 [11]. The 2005 ISUP modified Gleason system is considered as the currently accepted version of Gleason grading [12, 13].

In this study, we evaluated discrepancies in Gleason grading between urological pathologists and general pathologists. We also sought to evaluate the impact of Gleason grading by general pathologists.

## Methods

Between April 2006 and March 2008, we enrolled 755 patients who underwent prostate needle biopsy at 2 hospitals affiliated to the Nara Medical University. Approval for the study was obtained from the Nara Medical University Hospital Institutional Review Board. We obtained written informed consent from each enrolled patient before biopsy. Prostate-specific antigen (PSA) levels were determined using the PSA age-specific reference range according to Ito et al. [14] The cutoff value was 3.1 ng/mL in patients aged <65 years, 3.6 ng/mL in those aged 65–70 years, and 4.1 ng/mL in those aged ≥70 years. Biopsy was performed under transrectal ultrasonography while adjusting the number (6–12 cores) on the basis of the prostate volume and age (Table 1) [14, 15]. All general pathologists in both affiliated hospitals evaluated the biopsy samples.

**Table 1:** Optimal number of biopsy cores based on patient age and total prostate volume

Prostate volume (mL)	Age (yrs)			
	<60	60-64	65-69	≥70
0-25	12	10	8	6
25-50	12	12	10	8
50<	12	12	12	10

All biopsy samples were subsequently sent to Nara Medical University, where a urological pathological diagnosis was made by 2 experts in prostate cancer diagnosis who were blinded to the general pathologists’ evaluations. Each slide was diagnosed by 2 urological pathologists. When discrepancy between urological pathologists, they discussed with the case and determined the final diagnosis. The results were then returned to the general pathologists who reviewed the results given by the urological pathologist. The results were only described about GS, high grade prostatic intraepithelial neoplasia (HGPIN), atypical small acinar proliferation (ASAP), prostatitis, hypertrophy, or no malignancy (NM), and portion of cancer in each core. This procedure was followed for all samples. We compared the diagnostic accuracy between general and urological pathologists for the parameters of no malignancy, ASAP, HGPIN, and GS (6, 3 + 4, 4 + 3 and 8–10) at the worst GS for each patient. We then evaluated the concordance rate between general and urological pathologists for each 6-month period.

We used the Kruskal–Wallis test or chi-square test to estimate the distribution of each parameter in each term. The concordance was measured on the basis of the percentage of concordance and Cohen’s Kappa. Kappa values of 0.00–0.20, 0.21–0.40, 0.41–0.60, 0.61–0.80, and 0.81–1.00 represented slight, fair, moderate, substantial, and almost perfect concordance, respectively. These statistical analyses were performed using SPSS®, version 19 (SPSS Inc., Chicago, IL). Improvement of concordance over the course of each period was estimated by the chi-square test for trend using Graph Pad Prism®, version 5.01 (Graph Pad Software, San Diego, CA). A p-value of <0.05 was considered to be significant.

## Results

Table 2 shows patient characteristics for each 6-month period. No significant dispersion was noted for age, PSA level, the number of biopsy cores, or GS between the 4 groups using the Kruskal–Wallis test or the chi-square test.

**Table 2:** Characteristics of patients

	Overall (*n* = 755)	Apr./06-Sep./06 (*n* = 197)	Oct./06-Mar./07 (*n* = 225)	Apr./07-Sep./07 (*n* = 185)	Oct./07-Mar./08 (*n* = 148)	*p* Value
Age (median: yrs)	71 (41–90)	71 (46–94)	72 (41–85)	71 (46–91)	71 (44–90)	0.61^a^
PSA (median: ng/mL)	7.6 (0.3-5490)	7.6 (0.3–475)	7.6 (0.58–196)	7.2 (0.57–5490)	7.7 (1.0–864)	0.52^a^
No. of cores (median)	10 (6–12)	10 (6–12)	10 (6–12)	10 (6–12)	10 (6–12)	0.08^a^
GS 6 (%)	14.6 (*n* = 110)	17.2 (*n* = 34)	13.3 (*n* = 30)	11.9 (*n* = 22)	16.2 (*n* = 24)	0.51^b^
GS 3 + 4 (%)	11.1 (*n* = 84)	9.1 (*n* = 18)	12.0 (*n* = 27)	11.4 (*n* = 27)	8.1 (*n* = 12)	0.13^b^
GS 4 + 3 (%)	8.1 (*n* = 61)	5.6 (*n* = 11)	8.9 (*n* = 20)	7.0 (*n* = 13)	11.5 (*n* = 17)	0.23^b^
GS 8–10 %	6.6 (*n* = 50)	8.6 (*n* = 17)	6.2 (*n* = 14)	5.9 (*n* = 11)	5.4 (*n* = 8)	0.64^b^

*PSA* Prostate specific antigen, *GS* Gleason score

^a^Kruskal-wallis test. ^b^chi-square test

In the first period, the overall concordance rate of urological pathologists and general pathologists was 71.8 % (140/198 samples; Table 3). The urological pathologists diagnosed NM in 103 samples, ASAP in 4 samples, HGPIN in 11 patients, and prostate cancer in 80 samples. For 99 of 103 samples (96.1 %) diagnosed with NM, 1 of 4 samples (25.0 %) diagnosed with ASAP and 9 of 11 samples (81.8 %) with HGPIN, the general and urological pathologists’ diagnoses were in agreement. For 38 of these 80 samples (47.5 %), the general and urological pathologists’ GS diagnoses were in agreement and the kappa value was 0.55. The general pathologists undergraded 35.1 % (27/80) samples and overgraded 18.1 % (14/80) samples (Table 4). The general pathologists diagnosed 120 patients with NM and 5 patients with ASAP. Nine patients of patients diagnosed with NM and 2 of patients with ASAP by general pathologists were diagnosed with prostate cancer by urological pathologists.

**Table 3:** Concordance of the diagnosis of a needle biopsy between urological and general pathologists (Apr./06- Sep./06)

General pathologist diagnosis	Urological pathologist diagnosis								
	NM	ASAP	HGPIN	5	6	3 + 4	4 + 3	8–10	Overall
NM	99	3	9	0	9	0	0	0	
ASAP	2	1	0	0	2	0	0	0	
HGPIN	1	0	2	0	0	0	0	0	
5	0	0	0	0	2	0	0	0	
6	1	0	0	0	19	10	0	1	
3 + 4	0	0	0	0	1	4	1	2	
4 + 3	0	0	0	0	1	1	2	1	
8–10	0	0	0	0	0	3	8	13	
	103	4	11	0	34	18	11	17	197
% Exact Concordance	96.1	25.0	18.1	0	55.9	22.2	9.1	76.4	71.8
% Undergrading by UPD vs GPD					38.2	55.5	18.1	23.5	35.1
% Overgrading by UPD vs GPD					5.9	22.2	72.7	0	18.1

*UPD* urological pathologist diagnosis, *GPD* general pathologist diagnosis, *NM* No malignancy, *ASAP* atypical small acinar proliferation suspicious, *HGPIN* high grade prostatic intraepithelial hyperplasia

**Table 4:** Comparison of concordance of the Gleason score between urological and general pathologists in 4 periods

	% Exact Concordance of GS					% Undergrading by UPD vs GPD	% Overgrading by UPD vs GPD	Kappa score
	Overall	6	3 + 4	4 + 3	8–10			
Apr./06–Sep./06 (*n* = 80)	47.5 (38/80)	55.9 (19/34)	22.2 (4/18)	9.1 (2/11)	76.4 (13/17)	35.1 (28/80)	18.1 (14/80)	0.55
Oct./06–Mar./07 (*n* = 91)	62.6 (57/91)	86.7 (26/30)	66.7 (18/27)	25.0 (5/20)	57.1 (8/14)	23.1 (21/91)	14.3 (13/91)	0.68
Apr./07–Sep./07 (*n* = 65)	76.9 (50/65)	72.3 (16/22)	80.9 (17/21)	53.8 (7/13)	90.9 (10/11)	11.9 (8/65)	14.1 (9/65)	0.81
Oct./07–Mar./08 (*n* = 61)	78.7 (48/61)	87.5 (21/24)	75.0 (9/12)	70.6 (12/17)	75.0 (6/8)	16.4 (10/61)	4.9 (3/61)	0.84

*UPD* urological pathologist diagnosis, *GPD* general pathologist diagnosis, *GS* Gleason score

In the second period, the overall concordance rate of urological and general pathologists was 79.8 % (168/225 samples; Table 5). The urological pathologists diagnosed NM in 126 samples, ASAP in 2 samples, HGPIN in 6 patients, and prostate cancer in 91 samples. For 118 of 126 samples (93.7 %) diagnosed with NM, 2 of 2 samples (100 %) diagnosed with ASAP and 1 of 6 samples (16.7 %) with HGPIN, the general and urological pathologists’ diagnoses were in agreement. For 57 of these 91 samples (62.6 %), the general and urological pathologists’ GS diagnoses were in agreement and the kappa value was 0.68. General pathologists undergraded 23.1 % (21/91) samples and overgraded 14.3 % (13/91) samples (Table 4). The general pathologists diagnosed 125 patients with NM and 13 patients with ASAP. Two patients of patients diagnosed with NM and 4 of patients with ASAP were diagnosed with prostate cancer by urological pathologists.

**Table 5:** Concordance of the diagnosis of a needle biopsy between urological and general pathologists (Oct./06–Mar./07)

General pathologist diagnosis	Urological pathologist diagnosis								
	NM	ASAP	HGPIN	5	6	3 + 4	4 + 3	8-10	Overall
NM	118	0	5	0	0	0	0	2	
ASAP	7	2	0	0	0	4	0	0	
HGPIN	0	0	1	0	0	0	0	0	
5	0	0	0	0	0	0	0	0	
6	1	0	0	0	26	3	0	0	
3 + 4	0	0	0	0	4	18	8	1	
4 + 3	0	0	0	0	0	1	5	3	
8–10	0	0	0	0	0	1	7	8	
	126	2	6	0	30	27	20	14	225
% Exact Concordance	93.6	100	16.7	0	86.7	66.7	25.0	57.1	79.1
% Undergrading by UPD vs GPD					0	25.9	40.0	42.9	23.1
% Overgrading by UPD vs GPD					13.3	7.4	35.0	0	14.3

*UPD* urological pathologist diagnosis, *GPD* General pathologist diagnosis, *NM* no malignancy, *ASAP* atypical small acinar proliferation suspicious, *HGPIN* high grade prostatic intraepithelial hyperplasia

In the third period, the overall concordance rate of urological and general pathologists was 89.7 % (166/185; Table 6). The urological pathologists diagnosed NM in 115 samples, ASAP in 1 sample, HGPIN in 2 patients, and prostate cancer in 65 samples. For 115 of 115 samples (100 %) diagnosed with NM, 1 of 1 sample (100 %) diagnosed with ASAP and 0 of 2 samples (0 %) with HGPIN, the general and urological pathologists’ diagnoses were in agreement. For 50 out of these 65 samples (76.9 %), the general and urological pathologists’ GS diagnoses were in agreement and the kappa value was 0.81. General pathologists undergraded 11.9 % (8/65) samples and overgraded 14.1 % (9/65) samples (Table 4). The general pathologists diagnosed 117 patients with NM and 2 patients with ASAP. No patient of patients diagnosed with NM and 1 of patients with ASAP were diagnosed with prostate cancer by urological pathologists.

**Table 6:** Concordance of the diagnosis of a needle biopsy between urological and general pathologists (Apr./07–Sep./07)

General pathologist diagnosis	Urological pathologist diagnosis								
	NM	ASAP	HGPIN	5	6	3 + 4	4 + 3	8–10	Overall
NM	115	0	2	0	0	0	0	0	
ASAP	0	1	0	0	1	0	0	0	
HGPIN	0	0	0	0	0	0	0	0	
5	0	0	0	0	0	0	0	0	
6	0	0	0	0	16	3	1	0	
3 + 4	0	0	0	0	5	17	2	0	
4 + 3	0	0	0	0	0	1	7	1	
8–10	0	0	0	0	0	0	3	10	
	115	1	2	0	22	21	13	11	185
% Exact Concordance	100	100	0	0	72.3	80.9	53.8	90.9	89.7
% Undergrading by UPD vs GPD					4.5	14.3	23.1	9.1	11.9
% Overgrading by UPD vs GPD					22.7	4.8	23.1	0	14.1

*UPD* urological pathologist diagnosis, *GPD* general pathologist diagnosis, *NM* no malignancy, *ASAP* atypical small acinar proliferation suspicious, *HGPIN* High grade prostatic intraepithelial hyperplasia

In the last period, the overall concordance rate of urological and general pathologists was 89.9 % (133/148; Table 7). The urological pathologists diagnosed NM in 85 samples, ASAP in 1 sample, HGPIN in 1 sample, and prostate cancer in 61 samples. For 84 of 85 samples (98.8 %) diagnosed with NM, 1 of 1 sample (100 %) diagnosed with ASAP and 0 of 1 samples (0 %) with HGPIN, the general and urological pathologists’ diagnoses were in agreement. For 48 of these 61 samples (78.7 %), the general and urological pathologists’ GS diagnoses were in agreement and the kappa value was 0.84. General pathologists undergraded 16.4 % (10/61) samples and overgraded 4.9 % (3/61) samples (Table 4). The general pathologists diagnosed 86 patients with NM and 3 patients with ASAP. One patient of patients diagnosed with NM and 1 of patients with ASAP were diagnosed with prostate cancer by urological pathologists.

**Table 7:** Concordance of the diagnosis of a needle biopsy between urological and general pathologists (Oct./07–Mar./08)

General pathologist diagnosis	Urological pathologist diagnosis								
	NM	ASAP	HGPIN	5	6	3 + 4	4 + 3	8–10	Overall
NM	84	0	1	0	1	0	0	0	
ASAP	1	1	0	0	1	0	0	0	
HGPIN	0	0	0	0	0	0	0	0	
5	0	0	0	0	0	0	0	0	
6	0	0	0	0	21	3	0	0	
3 + 4	0	0	0	0	1	9	3	0	
4 + 3	0	0	0	0	0	0	12	2	
8–10	0	0	0	0	0	0	2	6	
	85	1	1	0	24	12	17	8	148
% Exact concordance	98.8	100	0	0	87.5	75.0	70.6	75.0	89.9
% Undergrading by UPD vs GPD					8.3	25.0	17.6	25.0	16.4
% Overgrading by UPD vs GPD					4.2	0	11.8	0	4.9

*UPD* Urological pathologist diagnosis, *GPD* General pathologist diagnosis, *NM* No malignancy, *ASAP* atypical small acinar proliferation suspicious, *HGPIN* High grade prostatic intraepithelial hyperplasia

The kappa value increased with time. The concordance rate significantly improved over the course of the study across periods (*p* = 0.04).

Fifty three patients were diagnosed with prostate cancer by urological pathologists on one positive core and 243 patients diagnosed with prostate cancer on two or more positive cores. Discrepancy between general and urological pathologists was found in 30 patients (56.6 %) of 53 and 76 patients (31.3 %) of 243 (p < 0.01), respectively.

## Discussion

Biopsy GS is an important predictor of the likelihood of various final pathological stages of radical retropubic prostatectomy [7], and it is also a significant predictor of biochemical recurrence in patients who undergo radical prostatectomy [16, 17]. Biopsy GS is also associated with biochemical failure in those who have undergone permanent brachytherapy [18] and external beam radiation therapy [19]. Biopsy GS, in combination with PSA level and clinical stage, is a very important factor in decision making for initial therapy. However, several studies have described interobserver variability in Gleason grading [8, 9]. In particular, the variability in Gleason grading between general pathologists should not be overlooked [8, 10]. Burchardt et al. demonstrated that 29 German pathologists who analyzed a series of tissue microarray images showed 45.7 % concordance with biopsy GS assigned by an expert. [20] Coard et al. reported 67 % overall concordance between anatomical pathologists and an experienced pathologist for consensus on prostate cancer GS [10]. In the present study, the overall concordance between general pathologists and the urological pathologists was 47.5 % and the kappa score was 0.55 in the first 6-month period. This was not an acceptable concordance and was similar to the results of previous studies. These discrepancies may have been caused by (1) a sampling effect caused by tumor heterogeneity, (2) interpretational bias, or (3) the small volume of tissue for cancer biopsy [10, 21]. In the present study, patients who diagnosed with prostate cancer on one positive core tended to be misdiagnosed compared to those who diagnosed on two or more cores and another reason for discordance may have been that the general pathologists did not refer to the 2005 ISUP consensus conference on the Gleason grading of Prostatic Carcinoma [11].

To improve this discrepancy, Mikami et al [22] used a 40-min educational lecture or a tutorial with an anatomical atlas. In a lecture group, the average concordance rates before and after the lecture were 55.7 % and 68.4 %, and the average kappa values were 0.43 and 0.67, respectively. In the atlas group, the average concordance rates before and after providing the atlas were 61.3 % and 74.5 %, and the average kappa values were 0.44 and 0.68, respectively. Allsbrook et al [8] reported that concordance between general pathologists and urological pathologists improved to 77.4 % (kappa value = 0.73) by web-based virtual microscopy. In Egevad’s study, the proportion of correct GS improved from 70.5 % to 86.6 % after a teaching set of 40 images illustrating GS was distributed among 85 pathologists [23]. The present study demonstrated an improvement in the accuracy of general pathologists’ GS after review by 2 urological pathologist. The rate of agreement and the kappa value increased with the period and improved from an initial 47.5 % (kappa score = 0.55) to a final 80.3 % (kappa value = 0.84). Furthermore in the third period, the rate of concordance was high and the high rate continued in the last period. So the appropriate time of this method for improving GS may need one year by our way.

It is well known that general pathologists tend to underestimate GS [8, 10, 20, 24]. Coard et al. reported that anatomical pathologists undergraded 25.6 % of all biopsy specimens and overgraded 6.7 % [10], whereas Burchardt et al. reported that the rates of undergrading and overgrading were 38.9 % and 15.4 %, respectively [20]. Similar to our reports, Barqawi et al. evaluated defference between outside pathologists and their institution pathologists and Gleason undergrading occurred in 46 % outside and 38 % their institution diagnosis with respect to radical prostatectomy specimens [24]. The corresponding values in the first period in the present study were 35.1 % and 18.1 %, respectively, showing that general pathologists tended to undergrade as in other reports. Undergrading was particularly common for tumors with a GS of 6 and 3 + 4 in our study. Allsbrook et al. found 47 % undergrading of tumors with GS 5–6, and 43 % undergrading of tumors with GS 7 [8]. In the present study, the undergrading of GS in 7 samples most probably resulted from mistaking Gleason pattern 4 for pattern 3, and the undergrading of GS in 6 tumors most probably resulted from mistaking pattern 3 for pattern 2. This is in accordance with the studies of Allsbrook et al. [8], Burchardt et al. [20], and Mikami et al. [22] Thus, there is a tendency for general pathologists to underestimate GS, especially in Gleason patterns 3 and 4.

However, the rate of undergrading decreased to 16.4 % in the last period after general pathologists had the experienced of review by the urological pathologists in the present study. Mikami et al. reported an improvement in the rate of undergrading from 36.3 % to 14.2 % after a lecture [22]. Egevad reported improvement of undergrading by the use of reference images [23]. It shows that the tendency for general pathologists to undergrade can improve when they study GS patterns using any of the common methods. Particular improvement in undergrading among general pathologists can be expected by preventing mistakes in identifying Gleason pattern 3 for pattern 2 and pattern 4 for pattern 3.

20 patients who diagnosed with NM and ASAP by general urologists were diagnosed with prostate cancer by urological patients. This discrepancy could be fatal. This discrepancy improved with time, 17/263 (6.5 %) in first and second period to 3/208 (1.4 %) in third and fourth period in the presents study (*p* = 0.01, chi-square test). Furthermore in 14 cases (70 %) the positive core was one. These result showed the discrepancy was caused by small cancer volume and interpretative error.

A limitation of this study was the inability to isolate the general pathologists from other educational sources associated with Gleason scoring over a period of 2 years. Therefore, any improvement seen may not necessarily be a direct result of the experience of the review by the urological pathologists.

## Conclusion

The concordance rate of GS between the urological and general pathologists was initially low (47.5 %), but following the expert reviews there was a significantly improvement in concordance rate over time.

## Abbreviations

ASAP: Atypical small acinar proliferation
GS: Gleason score, HGPIN, High grade prostatic intraepithelial neoplasia
ISUP: International Society of Urological Pathology
NM: No malignancy
PSA: Prostate-specific antigen
